# Effects of Early Intervention With Maternal Fecal Bacteria and Antibiotics on Liver Metabolome and Transcription in Neonatal Pigs

**DOI:** 10.3389/fphys.2019.00171

**Published:** 2019-02-27

**Authors:** Jia-Jia Wan, Chun-Hui Lin, Er-Du Ren, Yong Su, Wei-Yun Zhu

**Affiliations:** ^1^Laboratory of Gastrointestinal Microbiology, Jiangsu Key Laboratory of Gastrointestinal Nutrition and Animal Health, College of Animal Science and Technology, Nanjing Agricultural University, Nanjing, China; ^2^National Center for International Research on Animal Gut Nutrition, Nanjing Agricultural University, Nanjing, China

**Keywords:** fecal microbiota transplantation, liver, metabolite profile, neonatal piglet, transcriptional profile

## Abstract

The establishment of a stable bacterial flora in early life is associated with host metabolism. Studies of fecal microbiota transplantation (FMT) and antibiotics on neonatal pig mainly focused on intestinal development and mucosal immunity, but the information on metabolism is lacking. The objective of this study was to investigate the responses of metabolome and transcriptome in the livers of neonatal piglets that were orally inoculated with maternal fecal bacteria suspension and amoxicillin (AM) solution. Five litters of Duroc × Landrace × Yorkshire neonatal piglets were used as five replicates and nine piglets in each litter were randomly assigned to the control (CO), AM or FMT groups. Neonatal piglets in three groups were fed with 3 mL saline (0.9%), AM solution (6.94 mg/mL) or fecal bacteria suspension (>10^9^/mL), respectively, on days 1–6. At the age of 7 and 21 days, one piglet from each group in each litter was sacrificed, and the serum and liver were collected for analysis. The RNA sequencing analysis showed that the mRNA expressions of *arachidonate 12-lipoxygenase* (*ALOX12*)*, acetyl-CoA acyltransferase 2* (*ACAA2*)*, cytochrome P450 family 1 subfamily A member 2* (*CYP1A2*)*, glutamic–pyruvic transaminase 2* (*GPT2*) and *argininosuccinate synthase 1* (*ASS1*) were downregulated (*P* < *0.05*) by AM on day 7, and that the mRNA expressions of *arachidonate 15-lipoxygenase* (*ALOX15*)*, CYP1A2* and *GPT2* were downregulated (*P < 0.05*) by FMT on day 7. GC-MS analysis showed that AM and FMT treatments mainly affected fatty acid metabolism and amino acid metabolism on days 7 and 21. AM and FMT both reduced (*P < 0.05*) the blood levels of triglycerides and low density lipoprotein cholesterol (LDL-C) on day 7. AM reduced *(P < 0.05*) the blood level of cholesterol on day 21, and FMT reduced the blood levels of cholesterol, triglycerides and LDL-C on day 21. These results indicate that early intervention with FMT or AM can reduce fatty acid oxidative catabolism and amino acid biosynthesis of neonatal piglets, which provides a reference for regulation host metabolism through early intervention in animal production and even human health.

## Introduction

Animal intestinal microbiota has a great influence on the health and growth of its host, because they can provide nutrition, improve immune system and modulate gastrointestinal development for host (Diao et al., 2016). It was always considered that the intestines of the neonate are sterile and microbiota begins to colonize after delivery (Fanaro et al., 2003). A study reported that the reprogramming of gut microbiota in early life is beneficial for host metabolism later (Bäckhed, 2011). And the gut flora of neonate affected by cesarean has been association with development of type I diabetes, celiac disease and obesity (Decker et al., 2011; Rodríguez et al., 2015). Therefore, the establishment of a stable bacterial flora in early life has a connection with metabolism of host.

Amoxicillin (AM) is a broad-spectrum antibiotic that has a bactericidal effect through the inhibition of bacterial cell wall synthesis. Now antibiotics are widely concerned because of their resistance. A previous study showed that early antibiotic intervention could reduce *Escherichia coli* count, and decreased fecal amines concentration (Yu et al., 2017a). The early antibiotic intervention could affect the microbial colonization over a period of time, and also affect adaptive immune system (Schokker et al., 2015). Moreover, previous study revealed the early antibiotic intervention increased bacterial fermentation of aromatic amino acids and the expression of proinflammatory cytokines (Zhang et al., 2017). However, current studies on the effect of antibiotics on neonatal pigs are mainly focused on intestinal development and mucosal immunity, and research on the extraintestinal level is lacking.

Fecal microbiota transplantation (FMT) is a type of intervention therapy in which the functional flora of a healthy body is transplanted into a patient’s gastrointestinal tract and the intestinal microflora with normal function are reconstructed. FMT have been treated as an effective treatment for enteritis. Studies showed the significant therapeutic effect of FMT on pseudomembranous enterocolitis (Eiseman et al., 1958; Bowden et al., 1981) and relapsed *Clostridium difficile* enteritis (Hamilton et al., 2012). What is more, it was reported that intervention of FMT altered the intestinal microbiota structure and improved intestinal morphology and mucosal barrier of neonatal pigs (Hu et al., 2018), and could partially convey host gut characteristics from pigs to neonatal mice (Diao et al., 2016). While most studies focused on intestinal microflora and intestinal development, information on the roles of FMT in the body metabolism is not well described.

As the metabolic center, the liver is able to reflect the regulating effect of AM and fecal bacterial suspensions on the organism metabolism. In the present study, we hypothesized that the early intervention of AM and fecal bacteria suspension could affect the liver metabolism of new-born piglets, thereby affecting the metabolism of the whole body. Because pigs have a high similarity with man in physiology and other clinical research fields (Koopmans and Schuurman, 2015), we choose pig as a model to test, which can better reflect the human status. Thus, the effect of early intervention with antibiotics and fecal bacteria suspensions on liver transcription and metabolome in new-born piglets were investigated in this study through transcriptional analysis and metabolites profile analysis based on gas chromatography-mass spectrometry (GC-MS).

## Materials and Methods

### Ethics Statement

The experiment was approved and conducted under the supervision of the Animal Care and Use Committee of Nanjing Agricultural University (Nanjing, Jiangsu province, China). All pigs were raised and maintained on a local commercial farm under the care of the Animal Care and Use Guidelines of Nanjing Agricultural University.

### Preparation of Fecal Bacteria Suspension

The preparation of FMT was adapted from a previous method (Hamilton et al., 2012). In brief, approximately 50 g fresh feces from a healthy sow that was not treated with antibiotics were mixed with 250 mL sterile 0.9% NaCl solution. The mixture was homogenated and filtered with sterile gauze. The solution was transferred to 10 mL freezing tubes which were filled with CO_2_, and centrifuged at 2000 rpm for 10 min. The supernatant liquid was mixed with 10% sterile glycerol, and stored at −80°C.

### Piglet Experiment Design and Sampling

Five litters of healthy neonatal pigs (Duroc × Landrace × Yorkshire) from a commercial farm were used as five replicates. Nine piglets in each litter were randomly assigned into the AM, FMT or CO groups, with three piglets in each group. On days 1–6, the piglets in the AM group and CO group were orally administrated once a day with 3 mL AM solution (6.94 mg/mL), and physiological saline (0.9% NaCl), respectively, while the same volume of fecal bacteria suspension (>10^9^/mL) was offered to the piglets in the FMT group. All pigs had access to breast milk and water *ad libitum* and had no other creep feed during the experiment. At the age of 7 and 21 days, one piglet per group in each litter was randomly selected and slaughtered. Blood sample (10 mL) was collected from the anterior vena cava using heparin tube. The supernatant was collected through centrifuging at 3000 rpm for 15 min, and stored at −28°C for chemical analysis. The liver tissues were collected and stored in liquid nitrogen until transcriptome and metabolome analysis.

### Serum Measurement

Glucose, cholesterol, triglyceride, low density lipoprotein cholesterol (LDL-C), high density lipoprotein cholesterol (HDL-C), alanine aminotransferase, aspartate aminotransferase, total protein, serum albumin, globulin and alkaline phosphatase in the serum of piglets were measured with an Olympus AU400 Automatic Biochemical Analyzer (Tokyo, Japan) according to the manufacturer’s instructions.

### RNA Extraction and Sequencing

In order to reduce the cost of the experiment, three biological replicates were randomly selected from the samples for sequencing. Total RNA was extracted from liver tissue with the RNAiso Plus Total RNA extraction reagent (Takara) following the manufacturer’s instructions, and the RNA integrity was detected with Agilent Bioanalyzer 2100 (Agilent Technologies, United States). After purification, mRNA was enriched through removing ribosomal RNA (rRNA) with Ribo-Zero rRNA removal beads, and then RNA was split into fragments using divalent cations. The fragments were converted into the first strand cDNA using SuperScript II reverse transcriptase, and the synthesis of second stand cDNA was executed with DNA polymerase I and RNase H. After terminal repair, addition of base and connection of adapters, the fragments were amplified to create final cDNA libraries by PCR.

The sequencing was performed applying Illumina HiSeq 2500 according to the manufacturer’s instructions. About 6 G reads were produced in each sample, and the Q20 value of each sample was higher than 90%. Thus, the samples were qualified for further analysis. The quality results of RNA and sequencing is presented in Supplementary Table S1. The raw sequencing reads were submitted to Sequencing Read Archive database under BioProject: PRJNAS18006.

### Sample Preparation for GC-MS Analysis

About 100 mg liver tissues were taken in the 2 mL centrifuge tube and added with 1 mL of 80% methanol which pre-cooled at −20°C and five steel balls. The tubes were placed in a high-flux organization grinding apparatus at 70 Hz for 1 min, added with 60 μL of 2-chloro-L-phenylalanine (0.2 mg/mL stock in methanol) and 60 μL of heptadecanoic acid (0.2 mg/mL stock in methanol) as an internal quantitative standard and vortexed for 30 s. The tubes were put into an ultrasonic machine for 30 min at room temperature, and then stew for 30 min on the ice. The tubes were centrifuged for 10 min at 14,000 rpm (4°C), and 0.8 mL of the supernatant was transferred into a new centrifuge tube for blow-drying by vacuum concentration. The samples were added with 60 μL of methoxyamine pyridine solution (15 mg/mL), vortexed for 30 s, and reacted for 120 min at 37°C. About 60 μL of BSTFA reagent (containing 1% chlorotrimethylsilane) was added into the mixture, and then reacted for 90 min at 37°C. The supernatant that was obtained by centrifuging mixture for 10 min at 12,000 rpm (4°C) was transferred to inspect bottle.

### GC-MS Analysis

Each derivative sample (1 μL) was injected by the Agilent 7683 autosampler (Agilent Technologies, Atlanta, GA, United States) into the Agilent 6890 GC system equipped with a fused-silica capillary column (10 m × 0.18 mm i.d.) and a chemically bonded 0.18 μm stationary phase (DB-5; J&W Scientific, Folsom, CA, United States). The carrier gas (helium) passed through column at a speed of 1.0 mL/min. The column temperature is kept at 70°C for two min, and then increased to 310°C at a speed of 30°C/min and kept for two min. The column effluent was guided by a transmission route into the ion source of the mass spectrometer. The temperature of the transmission line and the ion source was 250 and 200°C, respectively. With a rate of 30 spectra/s, the mass spectra were generated in mass range of 50–800 m/z.

### Real-Time PCR

The RNA was extracted from liver tissue with the RNAiso Plus Total RNA extraction reagent (Takara), and then 1 μg RNA of each sample was converted into cDNA with the Reverse Transcriptase Kit (Takara, Japan) following the manufacturer’s instructions. Real time PCR was performed on ABI PRISM 7300 sequence detection system (SDS, Foster City, CA, United States) using SYBR Premix DimerEraserTM Kit (Takara, Japan) following the manufacturer’s instructions. The mRNA expressions of *cytochrome P450 C42* (*CYP2C42*) (Thörn et al., 2011)*, cytochrome P450 family 1 subfamily A member 2* (*CYP1A2*) (Kojima et al., 2008)*, acetyl-CoA acyltransferase 2* (*ACAA2*) (Sodhi et al., 2014), *tyrosine aminotransferase* (*TAT*)*, argininosuccinate synthase 1* (*ASS1*) and glycine amidinotransferase (*GATM*) were determined to verify the reliability of RNA-seq. The primers for *TAT, ASS1* and *GATM* were designed by Primer-BLAST^1^. The primer sequences are listed in Supplementary Table S2. The results were normalized to the expression level of the β-actin gene (Pieper et al., 2012), and the fold change (FC) was calculated with the 2^−ΔΔCt^ method.

### Data Analysis

The date of serum measurement and real-time PCR were analyzed by SPSS v. 20 as a completely randomized design, and one litter was regarded as one experiment unit (*n* = 5). The significance of the difference among the three treatments was evaluated by one-way ANOVA. A *P* < 0.05 was considered significantly different.

The raw data of RNA-seq were converted into clean sequences by removing the sequencing adapter and the low-complexity sequences with Seqtk^2^. Clean reads were mapped to the reference pig genome (Sus scrofa 10.2) with Hisat2 (version: 2.0.4). Then, the mapped results were quantitatively analyzed by Stringtie (v, 1.3.0) (Pertea et al., 2015). To normalize the gene expression levels, the RNA-seq reads were converted to fragments per kilobase of exon model per million mapped reads (FPKM) (Mortazavi et al., 2008). The differentially expressed genes (DEGs) of the three groups (*n* = 3) was analyzed with the edgeR (Robinson et al., 2010) and the FC was computed on the basis of the FPKM value. The DEGs with FC value > 1.5 or <0.67 and *P* value < 0.05 were chosen to further analyze.

DAVID Bioinformatics Resources 6.8^3^ (accessed 4 Dec 2017) was used to conduct the Kyoto Encyclopedia of Genes and Genomes (KEGG) Pathway enrichment analysis and Gene Ontology (GO) enrichment analysis. KEGG database can reflect the pathways involved in differentially genes. The statistical method used was Fisher’s exact test, and the false discovery rate (FDR) value was used as the FDR correct method. The enrichment was considered significant when *P* value < 0.05.

The raw date obtained by GC-MS was converted into netCDF format by Agilent MSD Chemstation (Smith et al., 2006). The identification, filtration and alignment of peak was performed with the XCMS program package of R (v3.1.3), and retention time, intensity and mass to charge ratio was acquired. The metabolites were annotated with AMDIS program based on National Institute of Standards and Technology database and Wiley Registry database. The obtained data were normalized to heptadecanoic acid. The online analysis tool of SmartNuclide Corporation^4^ (accessed 4 Oct 2017) was used to conduct the multivariate analysis of the GC-MS data that was pre-processed and differential metabolite set enrichment analysis. The obtained data was processed by partial least squares discriminant analysis (PLS-DA) (Supplementary Figure S1) (Szymańska et al., 2012). To reflect the quality of the model, R2Xcum, R2Ycum and Q2cum were calculated. The metabolites are selected to discriminate among the AM, CO and FMT group in accordance with this threshold of variable importance projection values > 1 and *P* values < 0.05. A hypergeometric test was used to conduct metabolite set enrichment analysis which affected by AM and fecal bacteria suspension.

## Results

### Serum Metabolites

On day 7, compared with the CO group, AM increased the concentrations of aspartate aminotransferase and alanine aminotransferase and decreased the concentrations of triglyceride and LCL-C (*P* < 0.05). FMT increased the concentrations of cholesterol, aspartate aminotransferase and alanine aminotransferase but decreased the levels of triglyceride and LDL-C (*P* < 0.05). Compared with the AM group, FMT increased the concentration of cholesterol but decreased the level of aspartate aminotransferase (*P* < 0.05). On day 21, AM increased the concentrations of aspartate aminotransferase and alanine aminotransferase, and decreased the level of cholesterol (*P* < 0.05). FMT increased the concentration of glucose but decreased the levels of cholesterol, triglyceride and LDL-C (*P* < 0.05). Compared with the AM group, FMT decreased the levels of LDL-C and aspartate aminotransferase (*P* < 0.05) (Supplementary Table S3).

### Metabolomic Analysis of the Liver

**FIGURE 1:**
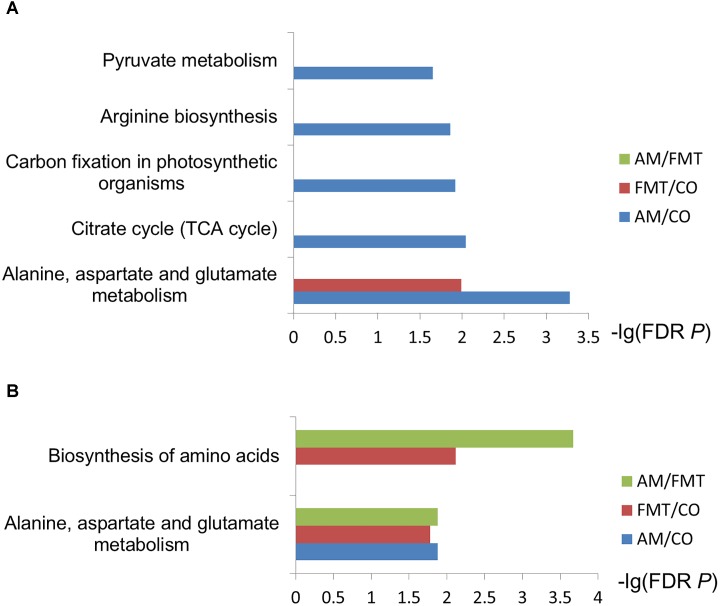
Enrichment analysis of selected metabolites. The metabolites discriminated among the amoxicillin (AM), fecal microbiota transplantation (FMT) and control (CO) groups on day 7 **(A)** and day 21 **(B)** were used for the metabolite set enrichment analysis based on the online analysis tool of Smart Nuclide Corporation. The rectangle color represents contrast, and the rectangle length is the false discovery rate-adjusted *P*-value.

On day 7, AM enriched 7 type of fatty acids and 3 type of amino acids (*P* < 0.05); FMT enriched 4 type of fatty acids and 3 type of amino acids (*P* < 0.05). Compared with AM, FMT decreased 3 type of fatty acids (*P* < 0.05) (Table 1). In the further metabolic pathway enrichment analysis, AM affected (FDR < 0.05) alanine, aspartate and glutamate metabolism, pyruvate metabolism, citrate cycle and arginine biosynthesis (Figure 1A). FMT affected (FDR < 0.05) alanine, aspartate and glutamate metabolism. No difference in metabolic pathways was found between the AM and FMT groups.

**Table 1:** Differential metabolites in pig livers among the amoxicillin (AM), fecal microbiota transplantation (FMT) and control (CO) groups on day 7.

	Metabolites	Biological roles	Metabolic subpathway	FC^1^	VIP^2^	*P*-value^3^
AM/CO	Glutamine	Amino acid	Arginine biosynthesis	3.34	1.464	0.007
	Asparagine	Amino acid	Alanine, aspartate and glutamate metabolism	1.81	1.355	0.016
	Ornithine	Amino acid	Ornithine cycle	1.61	1.417	0.011
	9,12-(Z,Z)-Octadecadienoic acid	Unsaturated fatty acids	Linoleic acid metabolism	1.38	1.73	<0.001
	9-(Z)-Octadecenoic acid	Unsaturated fatty acids	Fatty acid biosynthesis	1.35	1.77	<0.001
	Arachidonic acid	Unsaturated fatty acids	Arachidonic acid metabolism	1.23	1.618	0.003
	9-(Z)-Hexadecenoic acid	Unsaturated fatty acids	Fatty acid biosynthesis	1.17	1.416	0.012
	Hexadecanoic acid	Saturated fatty acids	Fatty acid biosynthesis	1.24	1.667	0.001
	Octadecanoic acid	Saturated fatty acids	Fatty acid biosynthesis	1.19	1.699	<0.001
	Heptanoic acid	Saturated fatty acids	Others	1.15	1.493	0.004
	1-Monohexadecanoylglycerol	lipid	Others	1.24	1.772	<0.001
	1-Monooctadecanoylglycerol	lipid	Others	1.12	1.517	0.009
	Pyruvic acid	Organates/carboxylates	Pyruvate metabolism	0.788	1.75	<0.001
	Malic acid	Organates/carboxylates	TCA cycle	1.19	1.609	0.003
	Fumaric acid	Organates/carboxylates	TCA cycle	1.25	1.625	0.001
	Uridine	nucleoside	Pyrimidine metabolism	0.644	1.469	0.009
	Adenosine-5-monophosphate	nucleoside	Purine metabolism	0.505	1.727	<0.001
	Uridine-5-monophosphate	nucleoside	Pyrimidine metabolism	3.93	1.605	0.001
	myo-Inositol-1-phosphate	Carbohydrate	Inositol phosphate metabolism	0.877	1.695	<0.001
	Fructose	Carbohydrate	Fructose and mannose metabolism	0.834	1.284	0.026
	Phosphoric acid	Oxidative phosphorylation relatives	Oxidative phosphorylation	0.931	1.283	0.024
FMT/CO	Uridine-5-monophosphate	nucleoside	Pyrimidine metabolism	4.86	1.964	<0.01
	Adenosine-5-monophosphate	nucleoside	Purine metabolism	0.277	1.936	<0.001
	Glutamine	Amino acid	Arginine biosynthesis	4.83	1.69	0.001
	Asparagine	Amino acid	Alanine, aspartate and glutamate metabolism	2.29	1.662	0.003
	S-methyl-Cysteine	Amino acid	Others	1.46	1.317	0.035
	Octadecanoic acid	Saturated fatty acids	Fatty acid biosynthesis	1.27	1.944	<0.001
	Hexadecanoic acid	Saturated fatty acids	Fatty acid biosynthesis	1.22	1.871	0.001
	9,12-(Z,Z)-Octadecadienoic acid	Unsaturated fatty acids	Linoleic acid metabolism	1.22	1.612	0.011
	9-(Z)-Octadecenoic acid	Unsaturated fatty acids	Fatty acid biosynthesis	1.19	1.536	0.018
	1-Monooctadecanoylglycerol	lipid	Others	1.14	1.33	0.023
	1-Monohexadecanoylglycerol	lipid	Others	1.1	1.418	0.031
	Xylose	Carbohydrate	Amino sugar and nucleotide sugar metabolism	0.877	1.412	0.026
	Galactose	Carbohydrate	Galactose metabolism	0.867	1.336	0.043
	Glucose	Carbohydrate	Glycolysis or Gluconeogenesis	0.867	1.336	0.043
	myo-Inositol-1-Phosphate	Carbohydrates	Inositol phosphate metabolism	0.801	1.92	<0.001
	1,3-Di-tert-butylbenzene	Aromatic compounds	Others	0.915	1.433	0.028
	Phosphoric acid	Oxidative phosphorylation relatives	Oxidative phosphorylation	0.908	1.493	0.021
	Pyruvic acid	Organates/carboxylates	Pyruvate metabolism	0.828	1.773	0.002
FMT/AM	Adenosine	nucleoside	Purine metabolism	1.65	1.985	0.001
	Adenosine-5-monophosphate	nucleoside	Purine metabolism	0.548	1.975	0.001
	Glyceric acid-3-phosphate	lipid	Glycolysis or Gluconeogenesis	1.32	1.712	0.014
	1-Monohexadecanoylglycerol	lipid	Others	0.885	1.825	0.006
	myo-Inositol-1-Phosphate	Carbohydrate	Inositol phosphate metabolism	0.914	1.691	0.015
	myo-Inositol	Carbohydrate	Galactose metabolism	0.729	1.457	0.049
	9-(Z)-Hexadecenoic acid	Unsaturated fatty acids	Fatty acid biosynthesis	0.892	1.535	0.040
	9-(Z)-Octadecenoic acid	Unsaturated fatty acids	Fatty acid biosynthesis	0.88	1.484	0.050
	Oxalic acid	carboxylates	Glyoxylate and dicarboxylate metabolism	0.945	1.465	0.046
	1,3-Di-tert-butylbenzene	Aromatic compounds	Others	0.935	1.588	0.022
	Heptanoic acid	Saturated fatty acids	Others	0.877	1.852	0.004

*^1^FC = fold change for concentration of metabolites derived from AM, FMT and CO groups. ^2^VIP = variable importance projection. This value was got from PLS-DA model with a threshold of 1. ^3^The significance P-value was got from Wilcoxon rank sum test with a threshold of 0.05.*

On day 21, AM enriched 5 type of fatty acids and 3 type of amino acids (*P* < 0.05); FMT enriched 5 type of fatty acid and 5 type of amino acid (*P* < 0.05). Compared with AM, FMT enriched 1 type of fatty acid and 5 type of amino acids (*P* < 0.05) (Table 2). Metabolic pathway enrichment analysis showed that AM affected (FDR < 0.05) alanine, aspartate and glutamate metabolism (Figure 1B). FMT affected (FDR < 0.05) the biosynthesis of amino acids and alanine, aspartate and glutamate metabolism. Two metabolic pathways (biosynthesis of amino acids and metabolism of alanine, aspartate and glutamate) were different between the AM and FMT groups (FDR < 0.05).

**Table 2:** Differential metabolites in pig livers in the AM, FMT, and CO groups on day 21.

	Metabolites	Biological roles	Metabolic subpathway	FC^1^	VIP^2^	*P*-value^3^
AM/CO	Glutamine	Amino acid	Arginine biosynthesis	3.9	2.089	0.001
	S-methyl-Cysteine	Amino acid	Others	1.25	1.475	0.035
	Asparagine	Amino acid	Alanine, aspartate and glutamate metabolism	1.77	2.102	0.001
	Pyroglutamic acid	Amino acid	Glutathione metabolism	0.822	1.883	0.006
	Uridine-5-monophosphate	nucleoside	Pyrimidine metabolism	3.09	1.857	0.004
	Adenosine-5-monophosphate	nucleoside	Purine metabolism	0.305	1.843	0.009
	Arachidonic acid	Unsaturated fatty acids	Arachidonic acid metabolism	1.38	2.044	0.001
	9,12-(Z,Z)-Octadecadienoic acid	Unsaturated fatty acids	Linoleic acid metabolism	1.3	1.853	0.007
	9-(Z)-Octadecenoic acid	Unsaturated fatty acids	Fatty acid biosynthesis	1.25	1.739	0.014
	Octadecanoic acid	Saturated fatty acids	Fatty acid biosynthesis	1.24	1.849	0.007
	Hexadecanoic acid	Saturated fatty acids	Fatty acid biosynthesis	1.24	1.7	0.026
	2-Hydroxyglutaric acid	Organates/carboxylates	Others	0.789	1.72	0.013
	Pyruvic acid	Organates/carboxylates	Pyruvate metabolism	0.727	1.917	0.006
	Sorbitol-6-phosphate	Carbohydrate	Fructose and mannose metabolism	1.22	2.041	0.001
	Phosphoric acid	Oxidative phosphorylation relatives	Oxidative phosphorylation	0.893	1.508	0.048
FMT/CO	Glutamine	Amino acid	Arginine biosynthesis	8.6	1.865	<0.001
	Asparagine	Amino acid	Alanine, aspartate and glutamate metabolism	2.96	1.947	<0.001
	Methionine	Amino acid	Cysteine and methionine metabolism	1.59	1.566	0.017
	Ornithine	Amino acid	Arginine biosynthesis	1.56	1.429	0.033
	S-methyl-Cysteine	Amino acid	Others	1.38	1.48	0.019
	Arachidonic acid	Unsaturated fatty acids	Arachidonic acid metabolism	1.56	1.737	0.003
	9,12-(Z,Z)-Octadecadienoic acid	Unsaturated fatty acids	Linoleic acid metabolism	1.49	1.672	0.006
	9-(Z)-Octadecenoic acid	Unsaturated fatty acids	Fatty acid biosynthesis	1.44	1.594	0.013
	Hexadecanoic acid	Saturated fatty acids	Fatty acid biosynthesis	1.44	1.758	0.003
	Octadecanoic acid	Saturated fatty acids	Fatty acid biosynthesis	1.4	1.917	<0.001
	1-Monooctadecanoylglycerol	Lipid	Others	1.33	1.608	0.008
	Glyceric acid-3-phosphate	Lipid	Glycolysis or Gluconeogenesis	0.768	1.375	0.023
	Hypoxanthine	Nucleoside	Purine metabolism	2.04	1.57	0.013
	Adenosine-5-monophosphate	Nucleoside	Purine metabolism	0.122	1.679	0.002
	Sorbitol-6-phosphate	Carbohydrate	Fructose and mannose metabolism	1.24	1.853	<0.001
	Phosphoric acid	Oxidative phosphorylation relatives	Oxidative phosphorylation	0.864	1.407	0.018
	Pyruvic acid	Organates/carboxylates	Pyruvate metabolism	0.704	1.613	0.005
FMT/AM	Glutamine	Amino acid	Arginine biosynthesis	2.2	1.823	0.00426
	Asparagine	Amino acid	Alanine, aspartate and glutamate metabolism	1.68	1.92	0.00143
	Glycine	Amino acid	Glycine, serine and threonine metabolism	1.49	1.453	0.0348
	Methionine	Amino acid	Cysteine and methionine metabolism	1.45	1.635	0.0165
	Alanine	Amino acid	Alanine, aspartate and glutamate metabolism	1.28	1.506	0.0312
	Ribose	Carbohydrate	Pentose phosphate pathway	1.17	1.539	0.0287
	Maltose	Carbohydrate	Starch and sucrose metabolism	2.07	1.721	0.00998
	Galactose	Carbohydrate	Galactose metabolism	0.94	1.599	0.023
	Glucose	Carbohydrate	Glycolysis or Gluconeogenesis	0.94	1.599	0.023
	Adenosine	nucleoside	Purine metabolism	0.666	1.55	0.028
	Uridine-5-monophosphate	Nucleoside	Pyrimidine metabolism	0.662	1.674	0.0125
	Adenosine-5-monophosphate	Nucleoside	Purine metabolism	0.402	1.654	0.0183
	Octadecanoic acid	Saturated fatty acids	Fatty acid biosynthesis	1.13	1.518	0.0321
	1-Monooctadecanoylglycerol	Lipid	Others	1.17	1.548	0.0285

*^1^FC = fold change for concentration of metabolites derived from AM, FMT and CO groups. ^2^VIP = variable importance projection. This value was got from PLS-DA model with a threshold of 1. ^3^The significance P-value was got from Wilcoxon rank sum test with a threshold of 0.05.*

### Gene Expression Profile in the Liver

**FIGURE 2:**
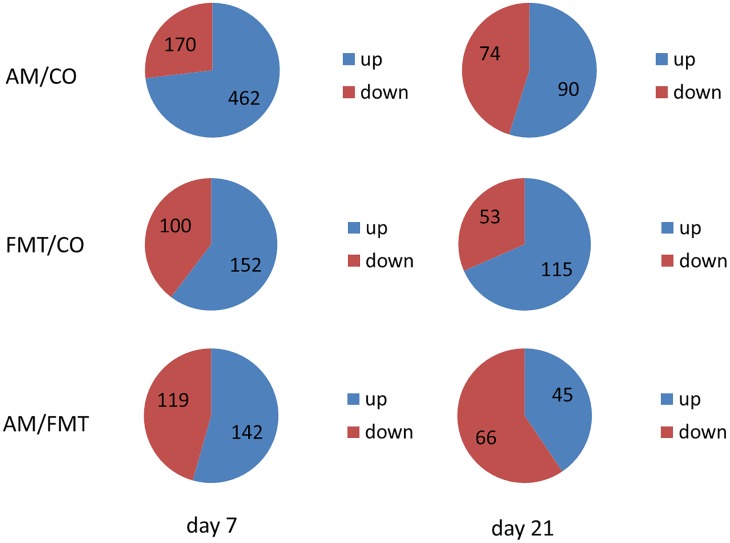
Number of the total differentially expressed genes (DEGs) as well as the up- and downregulated genes in the livers of pigs among the AM, FMT and CO groups on days 7 and 21.

On day 7, a total of 632 DEGs (*P* < 0.05) were found between the AM and CO groups, 252 DEGs (*P* < 0.05) between the FMT and CO groups and 261 DEGs (*P* < 0.05) between the AM and FMT groups (Figure 2). Among the DEGs, genes related to fatty acid metabolism, including *CYP2C42*, were upregulated, and *CYP1A2*, *arachidonate 12-lipoxygenase* (*ALOX12*) and *ACAA2* were downregulated (*P* < 0.05) by AM. FMT upregulated (*P < 0.05*) *CYP2C42* and downregulated (*P < 0.05*) *arachidonate 15-lipoxygenase* (*ALOX15*) and *CYP1A2* (Table 3). The amino acid metabolism related genes including *glutamic–pyruvic transaminase 2* (*GPT2*)*, arginase 1* (*ARG1*)*, TAT, ASS1, amine oxidase, copper containing 2* (*AOC2*)*, 5′-aminolevulinate synthase 2* (*ALAS2*)*, GATM and ornithine aminotransferase* (*OAT*), were downregulated by AM (*P* < 0.05), and FMT downregulated *GPT2* (Table 4). On day 21, a total of 164 DEGs (*P* < 0.05) were found between AM and CO, 168 DEGs (*P* < 0.05) between FMT and CO and 111 DEGs (*P* < 0.05) between AM and FMT (Figure 2). Among the DEGs, *ALOX15* was upregulated (*P* < 0.05) by AM (Table 3), and *ALAS2* was upregulated by AM and FMT (Table 4).

**Table 3:** Expression of fatty acid metabolism-related genes in the liver of piglets in the AM, FMT, and CO groups on days 7 and 21^1^.

Gene	Day 7			Day 21		
	AM/CO	FMT/CO	AM/FMT	AM/CO	FMT/CO	AM/FMT
CYP2C42	1.656(0.042)	1.955(0.000)	0.847(0.361)	1.174(0.529)	1.092(0.901)	1.075(0.702)
ALOX15	0.933(0.723)	0.330(0.001)	2.829(0.010)	3.564(0.025)	2.552(0.193)	1.397(0.395)
CYP1A2	0.457(0.005)	0.513(0.019)	0.890(0.297)	1.081(0.722)	1.075(0.835)	1.006(0.962)
ALOX12	0.603(0.002)	0.788(0.058)	0.765(0.124)	0.923(0.616)	0.952(0.624)	0.970(1)
ACAA2	0.600(0.007)	0.739(0.104)	0.813(0.105)	0.810(0.436)	0.926(0.514)	0.874(0.798)

*^1^Date were presented as fold change (P value); “0.000” means less than 0.001; The gene expressions were considered to be significantly altered when Fold change > 1.5 or < 0.67, P < 0.05; CYP2C42: cytochrome P450 C42; ALOX15: arachidonate 15-lipoxygenase; CYP1A2: cytochrome P450 family 1 subfamily A member 2; ALOX12: arachidonate 12-lipoxygenase; ACAA2: acetyl-CoA acyltransferase 2.*

**Table 4:** Expression of amino acid metabolism-related genes in the liver of piglets in the AM, FMT, and CO groups on days 7 and 21^1^.

Gene	Day 7			Day 21		
	AM/CO	FMT/CO	AM/FMT	AM/CO	FMT/CO	AM/FMT
GPT2	0.406(0.000)	0.566(0.007)	0.717(0.076)	1.200(0.389)	1.432(0.153)	0.838(0.494)
ARG1	0.541(0.000)	0.881(0.306)	0.614(0.005)	1.127(0.587)	1.162(0.613)	0.970(0.921)
TAT	0.599(0.003)	0.713(0.040)	0.840(0.242)	1.222(0.280)	0.905(0.422)	1.350(0.091)
ASS1	0.659(0.020)	0.771(0.165)	0.855(0.330)	1.051(0.859)	1.188(0.583)	0.884(0.707)
AOC2	0.585(0.004)	0.879(0.315)	0.665(0.119)	1.369(0.238)	1.279(0.357)	1.071(0.663)
ALAS2	0.609(0.017)	0.726(0.127)	0.838(0.326)	1.509(0.005)	1.895(0.040)	0.796(0.593)
GATM	0.618(0.035)	0.710(0.016)	0.870(0.488)	0.948(0.817)	0.669(0.105)	1.418(0.073)
OAT	0.455(0.000)	0.730(0.075)	0.624(0.016)	0.991(0.812)	1.160(0.666)	0.854(0.487)

*^1^Date were presented as fold change (P value); “0.000” means less than 0.001; The gene expressions were considered to be significantly altered when Fold change > 1.5 or < 0.67, P < 0.05; GPT2: glutamic–pyruvic transaminase 2; ARG1: arginase 1; TAT: tyrosine aminotransferase; ASS1: argininosuccinate synthase 1; AOC2: amine oxidase, copper containing 2; ALAS2: 5′-aminolevulinate synthase 2; GATM: glycine amidinotransferase; OAT: ornithine aminotransferase.*

The result of the GO enrichment analysis is presented in Supplementary Figure S2. On day 7, 24 terms were significantly varied. Most of terms were related to the metabolic process, genetic expression, immune process and cell process. Among them, AM significantly affected 19 of the terms (Supplementary Figure S2A). On day 21, a total of 23 terms were significantly varied (Supplementary Figure S2B). Most of the terms were related to the metabolic process, immune process, cell process, respond to stimulus and genetic expression.

**FIGURE 3:**
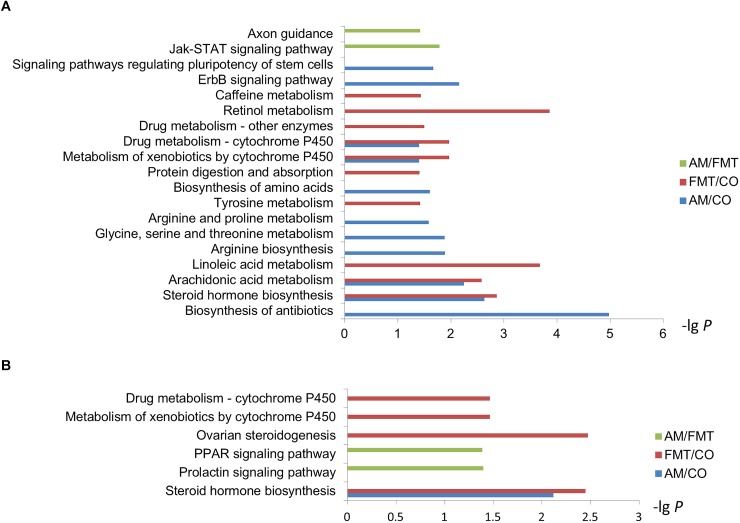
Kyoto Encyclopedia of Genes and Genomes pathway analysis of the DEGs in the livers among the AM, FMT and CO groups on day 7 **(A)** and day 21 **(B)**.

Kyoto Encyclopedia of Genes and Genomes enrichment analysis showed that on day 7, arachidonic acid metabolism and steroid hormone biosynthesis were significantly affected by AM and FMT treatment (Figure 3A). AM significantly affected the biosynthesis of antibiotics, arginine biosynthesis, biosynthesis of amino acids and arginine and proline metabolism and FMT significantly affected the retinol metabolism, linoleic acid metabolism and tyrosine metabolism. Compared with FMT, AM significantly affected axon guidance and Jak-STAT signaling pathway. On day 21, steroid hormone biosynthesis were significantly affected by AM and FMT. Only FMT significantly affected the ovarian steroidogenesis and relevant metabolism of cytochrome P450. Compared with FMT, AM significantly affected the prolactin signaling pathway and peroxisome proliferator-activated receptor (PPAR) signaling pathway (Figure 3B).

### Validation of the RNA-Seq Results by Quantitative PCR (qPCR)

**FIGURE 4:**
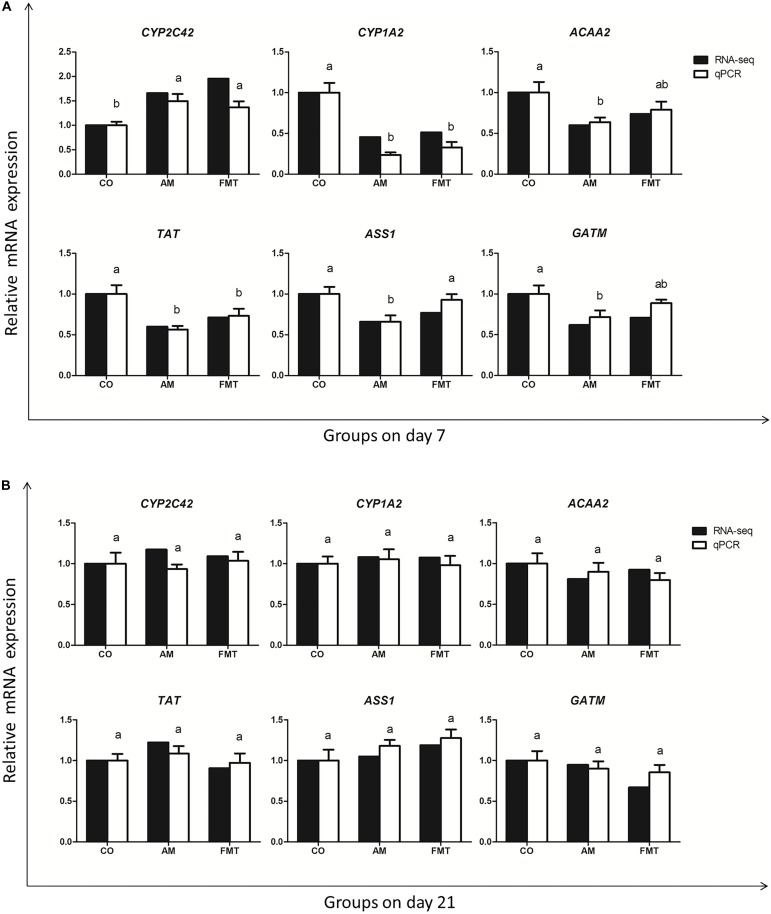
The qPCR validation of the RNA-seq. The results are displayed as the values of fold changes in the CO group on day 7 **(A)** and day 21 **(B)**, and the qPCR data are presented as the means ± SEM (*n* = 5). Values with different lowercase letter superscripts indicate a significant difference (*P* < 0.05), and those with the same letter superscripts indicate no significant difference (*P* > 0.05). CO: control; AM: amoxicillin; FMT: fecal microbiota transplantation. CYP2C42: cytochrome P450 C42; CYP1A2: cytochrome P450 family 1 subfamily A member 2; ACAA2: acetyl-CoA acyltransferase 2; TAT: tyrosine aminotransferase; ASS1: argininosuccinate synthase 1; GATM: glycine amidinotransferase.

To validate the gene expression profile from RNA-seq, we validated six genes (*CYP2C42, CYP1A2, ACAA2, TAT, ASS1 and GATM*) with qPCR. The qPCR dates of the genes are displayed in Supplementary Table S4. The result showed that the expression trend of these genes was the same as that detected by RNA-seq (Figure 4).

## Discussion

Antibiotics are the frontline therapy against microbial infectious diseases, but most antibiotics can cause side effects in humans (Singh et al., 2014). A previous study reported that amoxicillin–clavulanic acid could induce hepatitis characterized by elevated aminotransferase levels when people use amoxicillin–clavulanic acid for the treatment of chronic rhinosinusitis (Cundiff and Joe, 2007). In the current study, we found that piglets treated with AM had significantly elevated levels of aspartate aminotransferase and alanine aminotransferase compared with those in the CO group on days 7 and 21. Pefloxacin and AM significantly increased the concentration of cholesterol, triglyceride, HDL-C and LDL-C in the plasma of rats (Rotimi et al., 2015). Nonetheless, in the current study, the levels of triglyceride and LDL-C in the piglets treated with AM were significantly decreased on day 7, and the cholesterol level was also significantly decreased by AM on day 21. The differences in the effect of AM treatment could have resulted from the differences in species, age and environmental conditions of animals, as well as the dose of antibiotics used in the study. Interestingly, the FMT group had the same effect on triglyceride, LDL-C and cholesterol in plasma.

A previous study reported that subtherapeutic antibiotic treatment (penicillin VK, vancomycin, penicillin VK plus vancomycin and chlortetracycline) could significantly increase fat mass in mice and alter the fatty acid metabolism and lipid metabolic processes of the liver (Cho et al., 2012). In our study, we investigated the effects of antibiotics on the host hepatic transcriptome and metabolome in the pigs. AM significantly affected the KEGG of arachidonic acid metabolism on day 7, and the differential metabolite analysis revealed that AM increased seven types of fatty acids on day 7. Moreover, AM increased five types of fatty acids on day 21. Similarly, FMT significantly altered the linoleic acid and arachidonic acid metabolism in the KEGG term. It also increased four types of fatty acids on day 7 and five types of fatty acids on day 21. This phenomenon might suggest that FMT had a similar effect on the fatty acid metabolism to antibiotics.

Arachidonate 15-lipoxygenase encoded by *ALOX15* is capable of oxidizing polyenoic fatty acids, including arachidonic acid and linoleic acid (Banni et al., 2004; Wittwer and Hersberger, 2007; Martínez-Clemente et al., 2010; Ivanov et al., 2015). In our study, *ALOX15* was downregulated by FMT on day 7. Arachidonate 12-lipoxygenase encoded by *ALOX12* catalyzed the transformation of arachidonic acid into 12(S)-hydroperoxyeicosatetraenoic acid by oxygenating its 12-position (Liu et al., 2010). We found that *ALOX12* was downregulated by AM on day 7. Meanwhile, arachidonic acid was enriched by both AM and FMT treatments on days 7 and 21, and arachidonic acid metabolism were significantly affected by AM and FMT on day 7. This result suggests that both FMT and antibiotic can reduce oxidative catabolism of arachidonic acid.

The medium chain 3-ketoacyl-CoA thiolase encoded by *ACAA2* is essential to mitochondrial fatty acid β-oxidation (Houten and Wanders, 2010). *ACAA2* was downregulated by AM, and tended to be downregulated by FMT on day 7. This finding suggested that AM and FMT decreased fatty acid oxidation, such as arachidonic acid oxidation and linoleic acid oxidation. *CYP1A2* has been reported to catalyze the oxidative metabolism of polyunsaturated fatty acids, such as linoleic acid (Yao et al., 2006). However, *CYP1A2* was downregulated by AM and FMT in our study. And the differential metabolite analysis showed that multiple fatty acids in AM and FMT groups were significantly enriched. Thus, these results indicate that AM and FMT can increase the hepatic level of fatty acids by decreasing the fatty acid oxidation and catabolism.

Furthermore, previous studies revealed that antibiotics increased the serum concentration of most amino acids and decreased most amino acids of the small intestine, and that they supplemented the upregulation of the mRNA expression levels for key amino acid receptors and transporters of the small intestine (Puiman et al., 2013; Mu et al., 2017; Yu et al., 2017b). These findings suggested that antibiotics promoted amino acid absorption and significantly affected the metabolomics markers of amino acid metabolism in piglets (Hyde et al., 2003; Mu et al., 2017; Yu et al., 2017b). In our study, the KEGG analysis showed that AM significantly affected glycine, serine and threonine metabolism, arginine biosynthesis and arginine and proline metabolism on day 7. In the differential metabolic pathway analysis, AM significantly affected arginine biosynthesis and alanine, aspartate and glutamate metabolism on day 7 and alanine, aspartate and glutamate metabolism on day 21. Moreover, AM significantly increased three types of amino acids in the liver on day 7 and significantly altered four types of amino acids in the liver on day 21. Similarly, FMT had a significant effect on tyrosine metabolism. However, in metabolome analysis, FMT significantly altered the alanine, aspartate and glutamate metabolism on day 7 as well as the biosynthesis of amino acids and the metabolism of alanine, aspartate and glutamate on day 21. In addition, FMT significantly increased three type of amino acids on day 7 and five type of amino acids on day 21.

Glutamate pyruvate transaminase 2 encoded by *GPT2* catalyzes the reversible transference of an amino group from glutamate to pyruvate, yielding alanine and α-ketoglutarate (Ouyang et al., 2016). In this study, *GPT2* was downregulated by AM and FMT on day 7, and glutamine was enriched by AM and FMT on day 7 and 21. Meanwhile, pyruvic acid was decreased by AM and FMT. This phenomenon indicates that both AM and FMT may reduce transamination of glutamate to synthesize alanine and improve the alanine, aspartate and glutamate metabolism. Interestingly, AM also decreased the level of alanine in the liver on day 21 in comparison with the FMT treatment, which suggests that antibiotics have more significant effect on reducing alanine biosynthesis than FMT.

Argininosuccinate synthetase encoded by *ASS1* catalyzed the transformation of aspartate into arginine, which is described as the penultimate step of arginine biosynthesis (Haines et al., 2011; Lan et al., 2014), and *ASS1* was downregulated by AM in the present study. Asparagine in liver was enriched by AM and FMT, and the arginine biosynthesis pathway was significantly altered by AM on day 7. These findings suggest that AM can decrease arginine biosynthesis by reducing transformation of aspartate, and also increase the alanine, aspartate and glutamate metabolism.

The other genes related to amino acid metabolism, such as *ARG1*, *TAT, AOC2, ALAS2, GATM* and *OAT* were downregulated by AM on day 7. Compared with the FMT treatment, AM significantly affected the metabolic pathway of the biosynthesis of amino acids and the metabolism of alanine, aspartate and glutamate on day 21. This finding suggests that the impact of early intervention with antibiotics on decreasing the amino acid metabolism can last until day 21.

## Conclusion

In summary, our study investigated the transcriptome and metabolome responses induced by the short-term oral administration of AM and FMT in the early life of piglets. We found that both FMT and antibiotics could reduce fatty acid oxidative catabolism and amino acid biosynthesis in the liver, and that antibiotics had a more significant effect on reducing alanine biosynthesis and amino acid metabolism than FMT. Our finding provides a reference for regulation host metabolism through early intervention in animal production and even human health. However, more studies are needed for further understanding the impact and regulating mechanism of early intervention with AM and FMT on host metabolism in the later life.

## Author Contributions

YS and W-YZ conceived and designed the experiments. J-JW, C-HL, E-DR, and YS performed the experiments and analyzed the data. J-JW, C-HL, and YS wrote the paper.

## Conflict of Interest Statement

The authors declare that the research was conducted in the absence of any commercial or financial relationships that could be construed as a potential conflict of interest.

## Footnotes

1 https://www.ncbi.nlm.nih.gov/tools/primer-blast/index.cgi?LINK_LOC=BlastHome

2 https://github.com/lh3/seqtk

3 https://david.ncifcrf.gov/

4 http://www.biodeep.cn/

## References

[B1] Bäckhed F. (2011). Programming of host metabolism by the gut microbiota. *Ann. Nutr. Metabol.* 58 44–52. 10.1159/000328042 21846980

[B2] Banni S. Petroni A. Blasevich M. Carta G. Angioni E. Murru E. (2004). Detection of conjugated C16 PUFAs in rat tissues as possible partial beta-oxidation products of naturally occurring conjugated linoleic acid and its metabolites. *Biochim. Biophys. Acta* 1682 120–127. 10.1016/j.bbalip.2004.03.003 15158763

[B3] Bowden T. A. Mansberger A. R. Lykins L. E. (1981). Pseudomembraneous enterocolitis: mechanism for restoring floral homeostasis. *Am. Surg.* 47 178–183. 7224366

[B4] Cho I. Yamanishi S. Cox L. Methé B. A. Zavadil J. Li K. (2012). Antibiotics in early life alter the murine colonic microbiome and adiposity. *Nature* 488 621–626. 10.1038/nature11400 22914093 PMC3553221

[B5] Cundiff J. Joe S. (2007). Amoxicillin-clavulanic acid induced hepatitis. *Am. J. Otolaryngol.* 28 28–30. 10.1016/j.amjoto.2006.06.007 17162128

[B6] Decker E. Hornef M. Stockinger S. (2011). Cesarean delivery is associated with celiac disease but not inflammatory bowel disease in children. *Gut Microbes* 2 91–98. 10.4161/gmic.2.2.15414 21637025

[B7] Diao H. Yan H. L. Xiao Y. Yu B. Yu J. He J. (2016). Intestinal microbiota could transfer host gut characteristics from pigs to mice. *BMC Microbiol.* 16:253. 10.1186/s12866-016-0879-0 27729007 PMC5057279

[B8] Eiseman B. Silen W. Bascom G. S. Kauvar A. J. (1958). Fecal enema as an adjunct in the treatment of pseudomembranous enterocolitis. *Surgery* 44 854–859. 13592638

[B9] Fanaro S. Chierici R. Guerrini P. Vigi V. (2003). Intestinal microflora in early infancy: composition and development. *Acta Paediatr.* 91 48–55. 10.1111/j.1651-2227.2003.tb00646.x 14599042

[B10] Haines R. J. Pendleton L. C. Eichler D. C. (2011). Argininosuccinate synthase: at the center of arginine metabolism. *Int. J. Biochem. Mol. Biol.* 2 8–23. 21494411 PMC3074183

[B11] Hamilton M. J. Weingarden A. R. Sadowsky M. J. Khoruts A. (2012). Standardized frozen preparation for transplantation of fecal microbiota for recurrent Clostridium difficile infection. *Am. J. Gastroenterol.* 107 761–767. 10.1038/ajg.2011.482 22290405

[B12] Houten S. M. Wanders R. J. (2010). A general introduction to the biochemistry of mitochondrial fatty acid β-oxidation. *J. Inherit. Metab. Dis.* 33 469–477. 10.1007/s10545-010-9061-2 20195903 PMC2950079

[B13] Hu L. Geng S. Li Y. Cheng S. Fu X. Yue X. (2018). Exogenous fecal microbiota transplantation from local adult pigs to crossbred newborn piglets. *Front. Microbiol.* 8:2663. 10.3389/fmicb.2017.02663 29375527 PMC5767267

[B14] Hyde R. Taylor P. M. Hundal H. S. (2003). Amino acid transporters: roles in amino acid sensing and signalling in animal cells. *Biochem. J.* 373 1–18. 10.1042/bj20030405 12879880 PMC1223487

[B15] Ivanov I. Kuhn H. Heydeck D. (2015). Structural and functional biology of arachidonic acid 15-lipoxygenase-1 (ALOX15). *Gene* 573 1–32. 10.1016/j.gene.2015.07.073 26216303 PMC6728142

[B16] Kojima M. Sekimoto M. Degawa M. (2008). A novel gender-related difference in the constitutive expression of hepatic cytochrome P4501A subfamily enzymes in Meishan pigs. *Biochem. Pharmacol.* 75 1076–1082. 10.1016/j.bcp.2007.10.030 18068149

[B17] Koopmans S. J. Schuurman T. (2015). Considerations on pig models for appetite, metabolic syndrome and obese type 2 diabetes: from food intake to metabolic disease. *Eur. J. Pharmacol.* 759 231–239. 10.1016/j.ejphar.2015.03.044 25814261

[B18] Lan J. Tai H. C. Lee S. W. Chen T. J. Huang H. Y. Li C. F. (2014). Deficiency in expression and epigenetic DNA Methylation of ASS1 gene in nasopharyngeal carcinoma: negative prognostic impact and therapeutic relevance. *Tumor Biol.* 35 161–169. 10.1007/s13277-013-1020-8 23897555

[B19] Liu P. Lu Y. Recker R. R. Deng H. W. Dvornyk V. (2010). ALOX12 gene is associated with the onset of natural menopause in white women. *Menopause J. North Am. Menopause Soc.* 17 152–156. 10.1097/gme.0b013e3181b63c68 20061896 PMC2927106

[B20] Martínez-Clemente M. Ferré N. Titos E. Horrillo R. González-Périz A. Morán-Salvador E. (2010). Disruption of the 12/15-lipoxygenase gene (Alox15) protects hyperlipidemic mice from nonalcoholic fatty liver disease. *Hepatology* 52 1980–1991. 10.1002/hep.23928 20967760

[B21] Mortazavi A. Williams B. A. McCue K. Schaeffer L. Wold B. (2008). Mapping and quantifying mammalian transcriptomes by RNA-Seq. *Nat. Methods* 5 621–628. 10.1038/nmeth.1226 18516045 PMC13303166

[B22] Mu C. Yang Y. Yu K. Yu M. Zhang C. Su Y. (2017). Alteration of metabolomic markers of amino-acid metabolism in piglets with in-feed antibiotics. *Amino Acids* 49 771–781. 10.1007/s00726-017-2379-4 28101652

[B23] Ouyang Q. Nakayama T. Baytas O. Davidson S. M. Yang C. Schmidt M. (2016). Mutations in mitochondrial enzyme GPT2 cause metabolic dysfunction and neurological disease with developmental and progressive features. *Proc. Natl. Acad. Sci. U.S.A.* 113 E5598–E5607. 10.1073/pnas.1609221113 27601654 PMC5035873

[B24] Pertea M. Pertea G. M. Antonescu C. M. Chang T. C. Mendell J. T. Salzberg S. L. (2015). StringTie enables improved reconstruction of a transcriptome from RNA-seq reads. *Nat. Biotechnol.* 33 290–295. 10.1038/nbt.3122 25690850 PMC4643835

[B25] Pieper R. Kröger S. Richter J. F. Wang J. Martin L. Bindelle J. (2012). Fermentable fiber ameliorates fermentable protein-induced changes in microbial ecology, but not the mucosal response, in the colon of piglets. *J. Nutr.* 142 661–667. 10.3945/jn.111.156190 22357743

[B26] Puiman P. Stoll B. Mølbak L. Bruijn A. D. Schierbeek H. Boye M. (2013). Modulation of the gut microbiota with antibiotic treatment suppresses whole body urea production in neonatal pigs. *Am. J. Physiol. Gastrointest. Liver Physiol.* 304 G300–G310. 10.1152/ajpgi.00229.2011 23139222 PMC3566514

[B27] Robinson M. D. McCarthy D. J. Smyth G. K. (2010). edgeR: a Bioconductor package for differential expression analysis of digital gene expression data. *Bioinformatics* 26 139–140. 10.1093/bioinformatics/btp616 19910308 PMC2796818

[B28] Rodríguez J. M. Murphy K. Stanton C. Ross R. P. Kober O. I. Juge N. (2015). The composition of the gut microbiota throughout life, with an emphasis on early life. *Microb Ecol Health Dis.* 26:26050. 10.3402/mehd.v26.26050 25651996 PMC4315782

[B29] Rotimi S. O. Ojo D. A. Talabi O. A. Ugbaja R. N. Balogun E. A. Ademuyiwa O. (2015). Amoxillin- and pefloxacin-induced cholesterogenesis and phospholipidosis in rat tissues. *Lipids Health Dis.* 14 13–30. 10.1186/s12944-015-0011-8 25879817 PMC4339583

[B30] Schokker D. Jansman A. Bruin N. D. Vastenhouw S. Bree F. D. Bossers A. (2015). Impact on gut development of an early life oral antibiotic intervention in broilers. *Wageningen Livestock Res. Livestock Res. Rep.* 859 1–30.

[B31] Singh R. Sripada L. Singh R. (2014). Side effects of antibiotics during bacterial infection: mitochondria, the main target in host cell. *Mitochondrion* 16 50–54. 10.1016/j.mito.2013.10.005 24246912

[B32] Smith C. A. Want E. J. O’Maille G. Abagyan R. Siuzdak G. (2006). XCMS: processing mass spectrometry data for metabolite profiling using nonlinear peak alignment, matching, and identification. *Anal. Chem.* 78 779–787. 10.1021/ac051437y 16448051

[B33] Sodhi S. S. Ghosh M. Song K. D. Sharma N. Kim J. H. Kim N. E. (2014). An approach to identify SNPs in the gene encoding acetyl-CoA acetyltransferase-2 (ACAT-2) and their proposed role in metabolic processes in pig. *PLoS One* 9:e102432. 10.1371/journal.pone.0102432 25050817 PMC4106792

[B34] Szymańska E. Saccenti E. Smilde A. K. Westerhuis J. A. (2012). Double-check: validation of diagnostic statistics for PLS-DA models in metabolomics studies. *Metabolomics* 8 3–16. 10.1007/s11306-011-0330-3 22593721 PMC3337399

[B35] Thörn H. A. Lundahl A. Schrickx J. A. Dickinson P. A. Lennernäs H. (2011). Drug metabolism of CYP3A4, CYP2C9 and CYP2D6 substrates in pigs and humans. *Eur. J. Pharmaceut. Sci.* 43 89–98. 10.1016/j.ejps.2011.03.008 21447389

[B36] Wittwer J. Hersberger M. (2007). The two faces of the 15-lipoxygenase in atherosclerosis. *Prostaglandins Leukot. Essent. Fatty Acids* 77 67–77. 10.1016/j.plefa.2007.08.001 17869078

[B37] Yao H. T. Chang Y. W. Lan S. J. Chen C. T. Hsu J. T. Yeh T. K. (2006). The inhibitory effect of polyunsaturated fatty acids on human CYP enzymes. *Life Sci.* 79 2432–2440. 10.1016/j.lfs.2006.08.016 16978661

[B38] Yu M. Mu C. Yang Y. Zhang C. Su Y. Huang Z. (2017a). Increases in circulating amino acids with in-feed antibiotics correlated with gene expression of intestinal amino acid transporters in piglets. *Amino Acids* 49 1587–1599. 10.1007/s00726-017-2451-0 28623466

[B39] Yu M. Zhang C. Yang Y. Mu C. Su Y. Yu K. (2017b). Long-term effects of early antibiotic intervention on blood parameters, apparent nutrient digestibility, and fecal microbial fermentation profile in pigs with different dietary protein levels. *J. Anim. Sci. Biotechnol.* 8:60. 10.1186/s40104-017-0192-2 28781770 PMC5537924

[B40] Zhang C. Yu M. Yang Y. Mu C. Su Y. Zhu W. (2017). Differential effect of early antibiotic intervention on bacterial fermentation patterns and mucosal gene expression in the colon of pigs under diets with different protein levels. *Appl. Microbiol. Biotechnol.* 101 2493–2505. 10.1007/s00253-016-7985-7 27913852

