# Supplementary material for: Effects of Early Intervention With Maternal Fecal Bacteria and Antibiotics on Liver Metabolome and Transcription in Neonatal Pigs
Source: Front Physiol. 2019 Feb 27;10:171. doi: 10.3389/fphys.2019.00171 (PMC6413716; doi:10.3389/fphys.2019.00171)
Supplement: Supplementary file 1 [file Data_Sheet_1.doc]

**Journal name: Frontiers in Physiology**

**Effects of early intervention with maternal faecal bacteria and antibiotics on liver metabolome and transcription in neonatal pigs**

Jia-Jia Wan, Chun-Hui Lin, Er-Du Ren, Yong Su and Wei-Yun Zhu

**Supplementary Material**

***Table S1.*** *RNA and sequencing data quality control results*

| Sample^3^ | RIN^1^ | 28S/18S | Orientation | Raw reads | Clean reads | Q20^2^ Value |
| --- | --- | --- | --- | --- | --- | --- |
| A-7-1 | 9.4 | 1.7 | Forward/Reverse | 71,761,214 | 68,377,798 | 96.60 |
| A-7-2 | 9.6 | 2.1 | Forward/Reverse | 86,736,858 | 82,588,811 | 96.34 |
| A-7-3 | 9.5 | 1.8 | Forward/Reverse | 88,943,924 | 84,335,252 | 96.39 |
| A-21-1 | 7.4 | 1.6 | Forward/Reverse | 59,462,122 | 57,802,676 | 96.50 |
| A-21-2 | 7.9 | 1.7 | Forward/Reverse | 55,965,844 | 54,163,702 | 96.27 |
| A-21-3 | 7.4 | 1.7 | Forward/Reverse | 58,200,318 | 56,356,923 | 96.50 |
| S-7-1 | 8.3 | 1.7 | Forward/Reverse | 56,823,238 | 52,232,084 | 96.77 |
| S-7-2 | 9.1 | 1.9 | Forward/Reverse | 60,723,184 | 58,548,961 | 96.26 |
| S-7-3 | 8.5 | 1.7 | Forward/Reverse | 55,761,808 | 53,867,942 | 95.31 |
| S-21-1 | 7.7 | 1.8 | Forward/Reverse | 55,114,918 | 53,256,461 | 96.49 |
| S-21-2 | 7.2 | 1.7 | Forward/Reverse | 56,733,822 | 54,949,185 | 96.32 |
| S-21-3 | 7.8 | 1.8 | Forward/Reverse | 51,563,920 | 49,180,841 | 95.47 |
| F-7-1 | 8.0 | 1.7 | Forward/Reverse | 62,346,860 | 60,536,590 | 92.97 |
| F-7-2 | 8.5 | 2.1 | Forward/Reverse | 59,460,218 | 57,478,813 | 96.44 |
| F-7-3 | 8.6 | 1.8 | Forward/Reverse | 62,933,660 | 60,463,901 | 96.30 |
| F-21-1 | 8.3 | 1.4 | Forward/Reverse | 63,967,264 | 61,847,084 | 94.88 |
| F-21-2 | 8.4 | 1.4 | Forward/Reverse | 58,633,462 | 56,829,865 | 94.92 |
| F-21-3 | 8.9 | 1.7 | Forward/Reverse | 56,848,358 | 55,111,121 | 94.45 |

^1^RIN = RNA Integrity Number

^2^Q20 = bases of Q≥20 / all bases of sequencing

^3^ A = amoxicillin; S = control; F = faecal microbiota transplantation; 7 = day 7; 21 = day 21.

**Table S2. Primers lists used for real-time PCR assay in this study.^1^**

| Gene | Sequence 5’−3’ |
| --- | --- |
| *CYP2C42* | F: GCTGCTGTGCTGTCATCTGC |
|  | R: TCTAGTGGAGGTTGGGTGTTCA |
| *CYP1A2* | F: GTGAGGAGATGTTCAGCATCGTGAAG |
|  | R: CTTCTGTATCTCAGGATATGTCACA |
| *ACAA2* | F: TAGGCTCTGTGGCTCTGGTT |
|  | R: GTAATTGCCATCGGGATTTG |
| *TAT* | F: CCCAGGAATGTGCTTCGAGT |
|  | R: AGTGCTGCTCACAGAACTCC |
| *ASS1* | F: GGATGTCCAGCAAAGGCTCT |
|  | R: ACATGGCACTGGACTGGATG |
| *GATM* | F: GCCTCGAGACATCCTGATCG |
|  | R: CAGCCATTGTGGGCTTAGGA |
| β-actin | F: AGAGCGCAAGTACTCCGTGT |
|  | R: ACATCTGCTGGAAGGTGGAC |

^1^F: Forward, R: Reverse; CYP2C42: cytochrome P450 C42; CYP1A2: cytochrome P450 family 1 subfamily A member 2; ACAA2: acetyl-CoA acyltransferase 2; TAT: tyrosine aminotransferase; ASS1: argininosuccinate synthase 1; GATM: glycine amidinotransferase.

*Table S3. Serum metabolite concentrations of pig in amoxicillin (AM), faecal microbiota transplantation (FMT), and control (CO) groups1*

| Parameter^2^ | d 7 |  |  | d 21 |  |  |
| --- | --- | --- | --- | --- | --- | --- |
|  | AM | FMT | CO | AM | FMT | CO |
| Glucose (mmol/L) | 6.42(0.11)^a^ | 6.02(0.17)^ab^ | 5.97(0.11)^b^ | 7.20(0.30)^a^ | 7.15(0.24)^a^ | 6.34(0.15)^b^ |
| Cholesterol (mmol/L) | 4.51(0.15)^b^ | 5.28(0.15)^a^ | 4.51(0.06)^b^ | 3.23(0.30)^b^ | 3.09(0.25)^b^ | 4.09(0.26)^a^ |
| Triglyceride (mmol/L) | 1.37(0.07)^b^ | 1.54(0.08)^b^ | 1.75(0.05)^a^ | 0.97(0.05)^ab^ | 0.92(0.08)^b^ | 1.12(0.06)^a^ |
| HDL-C (mmol/L) | 1.60(0.06) | 1.65(0.04) | 1.49(0.07) | 1.66(0.17) | 1.58(0.09) | 1.55(0.08) |
| LDL-C (mmol/L) | 2.37(0.15)^b^ | 2.55(0.11)^b^ | 2.82(0.06)^a^ | 2.50(0.22)^a^ | 1.36(0.11)^b^ | 2.18(0.14)^a^ |
| AST (u/L) | 50.4(1.33)^a^ | 35.47(1.19)^b^ | 29.86(0.73)^c^ | 33.2(2.06)^a^ | 27.4(1.69)^b^ | 27(1.30)^b^ |
| ALT (u/L) | 30.6(0.68)^a^ | 28.34(0.38)^a^ | 24.8(0.59)^b^ | 25.2(1.36)^a^ | 23.2(0.73)^ab^ | 19.8(1.28)^b^ |
| TP (g/L) | 43.96(2.16)^c^ | 58.00(1.73)^a^ | 52.87(0.62)^b^ | 53.19(0.90) | 50.30(1.17) | 48.86(1.95) |
| ALB (g/L) | 14.14(0.28)^b^ | 16.89(0.40)^a^ | 16.36(0.21)^a^ | 27.78(0.69) | 27.72(0.82) | 26.84(0.85) |
| GLOB (g/L) | 27.56(1.32)^c^ | 41.86(0.51)^a^ | 36.25(1.12)^b^ | 24.90(1.22)^a^ | 21.28(0.43)^b^ | 22.84(0.94)^ab^ |
| ALP (u/L) | 1377.33(24.59)^b^ | 1426.8(19.81)^b^ | 1502.4(26.91)^a^ | 358.6(17.07)^ab^ | 330.2(13.33)^b^ | 399.8(18.66)^a^ |

^1^Data are presented as means (SEM). Values with different lowercase letter superscripts mean significant difference (*P* < 0.05), while with the same letter superscripts mean no significant difference (*P* > 0.05)

^2^ HDL-C=high-density lipoprotein cholesterol; LDL-C=and low-densitylipoprotein cholesterol; AST=Aspartate aminotransferase; ALT=Alanine aminotransferase; TP=Total protein; ALB=Serum albumin; GLOB=Globulin; ALP=alkaline phosphatase.

**Table S4.** Quantitative real-time PCR Ct values of the validated genes in the liver of pigs in the amoxicillin (AM), faecal microbiota transplantation (FMT) and control (CO) groups on day (d) 7 and day (d) 21.

|  | Genes^1^ | AM | | CO | | FMT | |
| --- | --- | --- | --- | --- | --- | --- | --- |
|  |  | Mean | SD | Mean | SD | Mean | SD |
| d 7 | CYP2C42 | 21.18 | 1.05 | 20.39 | 0.27 | 20.36 | 0.74 |
|  | CYP1A2 | 23.70 | 0.87 | 20.26 | 0.55 | 22.35 | 0.77 |
|  | ACAA2 | 23.18 | 0.92 | 21.20 | 0.73 | 21.95 | 1.05 |
|  | TAT | 20.11 | 1.12 | 17.95 | 0.69 | 18.82 | 0.95 |
|  | ASS1 | 19.82 | 1.28 | 17.86 | 0.57 | 18.37 | 0.78 |
|  | GATM | 20.93 | 1.42 | 19.08 | 0.73 | 19.64 | 0.77 |
| d 21 | CYP2C42 | 21.16 | 0.54 | 21.17 | 0.55 | 20.79 | 0.72 |
|  | CYP1A2 | 18.09 | 0.64 | 18.20 | 0.48 | 17.95 | 0.30 |
|  | ACAA2 | 19.28 | 0.53 | 19.19 | 0.27 | 19.19 | 0.58 |
|  | TAT | 18.47 | 0.61 | 18.64 | 0.20 | 18.40 | 0.17 |
|  | ASS1 | 17.66 | 0.46 | 17.99 | 0.51 | 17.31 | 0.47 |
|  | GATM | 18.13 | 0.47 | 18.04 | 0.36 | 17.97 | 0.34 |

^1^CYP2C42: cytochrome P450 C42; CYP1A2: cytochrome P450 family 1 subfamily A member 2; ACAA2: acetyl-CoA acyltransferase 2; TAT: tyrosine aminotransferase; ASS1: argininosuccinate synthase 1; GATM: glycine amidinotransferase**.**

**Figure S1.** Partial least squares discriminant analysis of the metabolites in the livers of pigs in the amoxicillin (AM), faecal microbiota transplantation (FMT) and control (CO) groups on days 7 and 21. PC1 = the first principal component; PC2 = the second principal component; R^2^X = the fraction of variance explained by a component; PC1 (23.3%) = PC1 explains 23.3% of the variation; PC2 (13.6%) = PC 2 explains 13.6% of the variation; PC1 (22.5%) = PC1 explains 22.5% of the variation; PC2 (14%) = PC2 explains 14% of the variation.


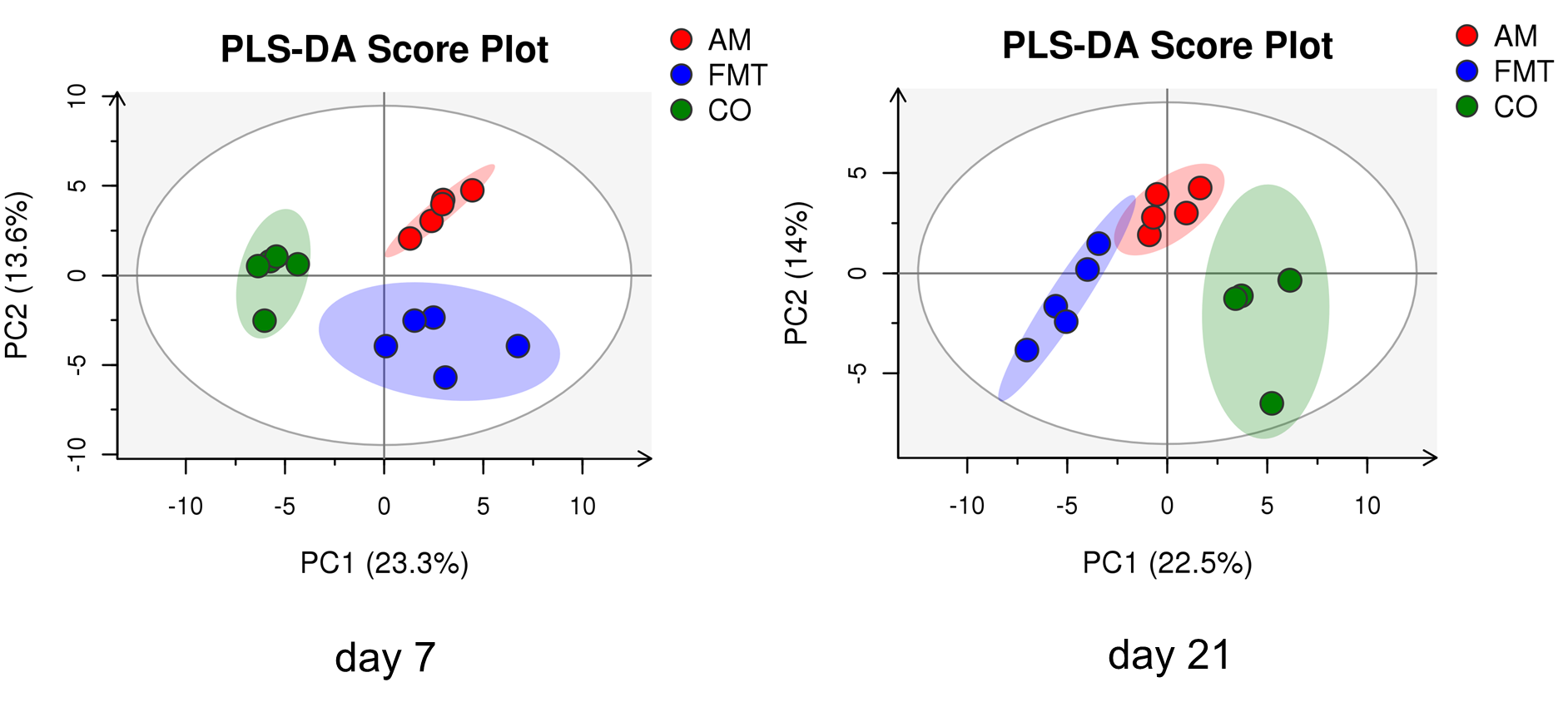


**Figure S2.** Gene ontology (GO) term enrichment analysis of the biological processes of the differentially expressed genes in the livers of pigs induced by the amoxicillin (AM), faecal microbiota transplantation (FMT) and control (CO) groups on days 7 (A) and 21 (B).

**A**

**
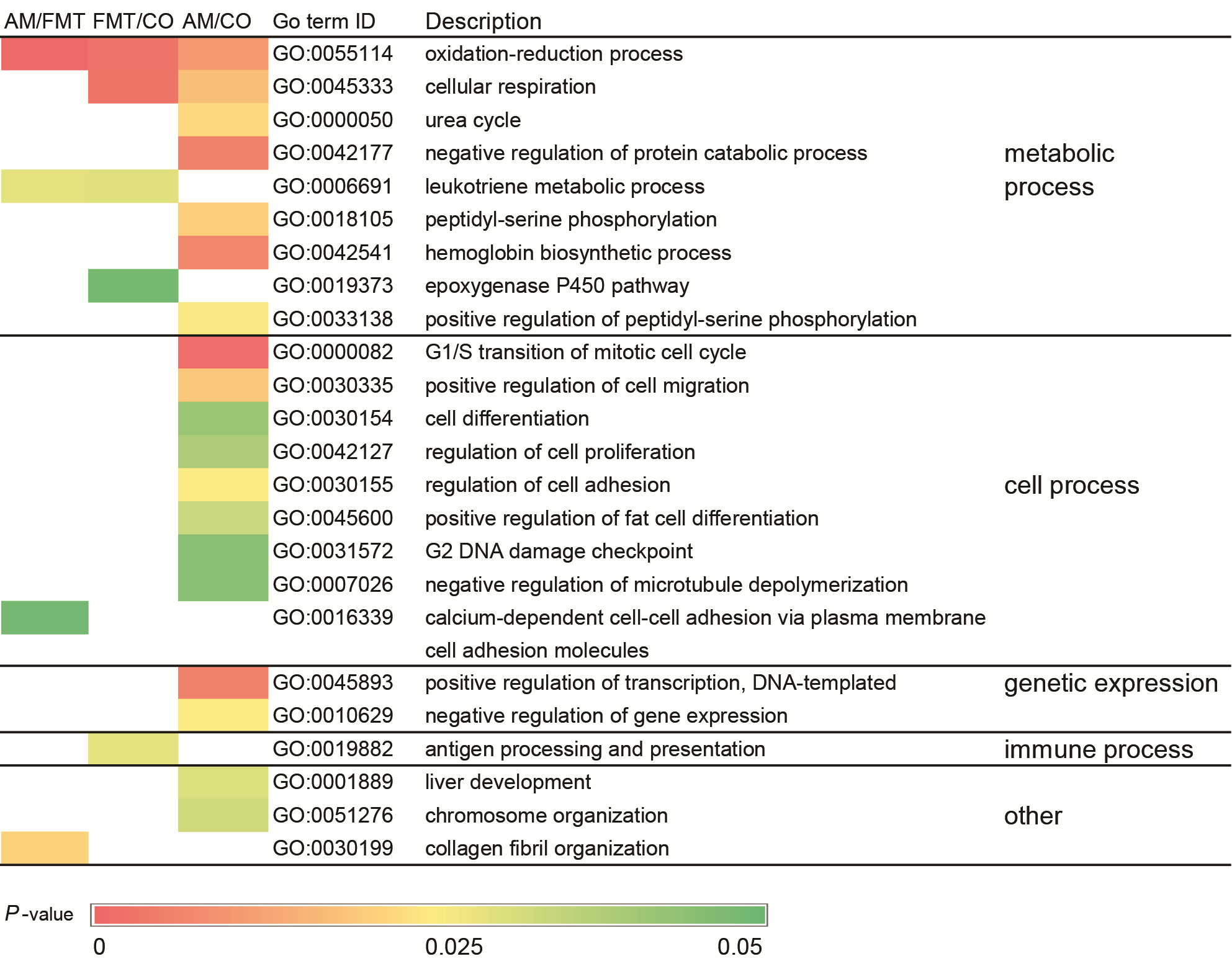
**

**B**

**
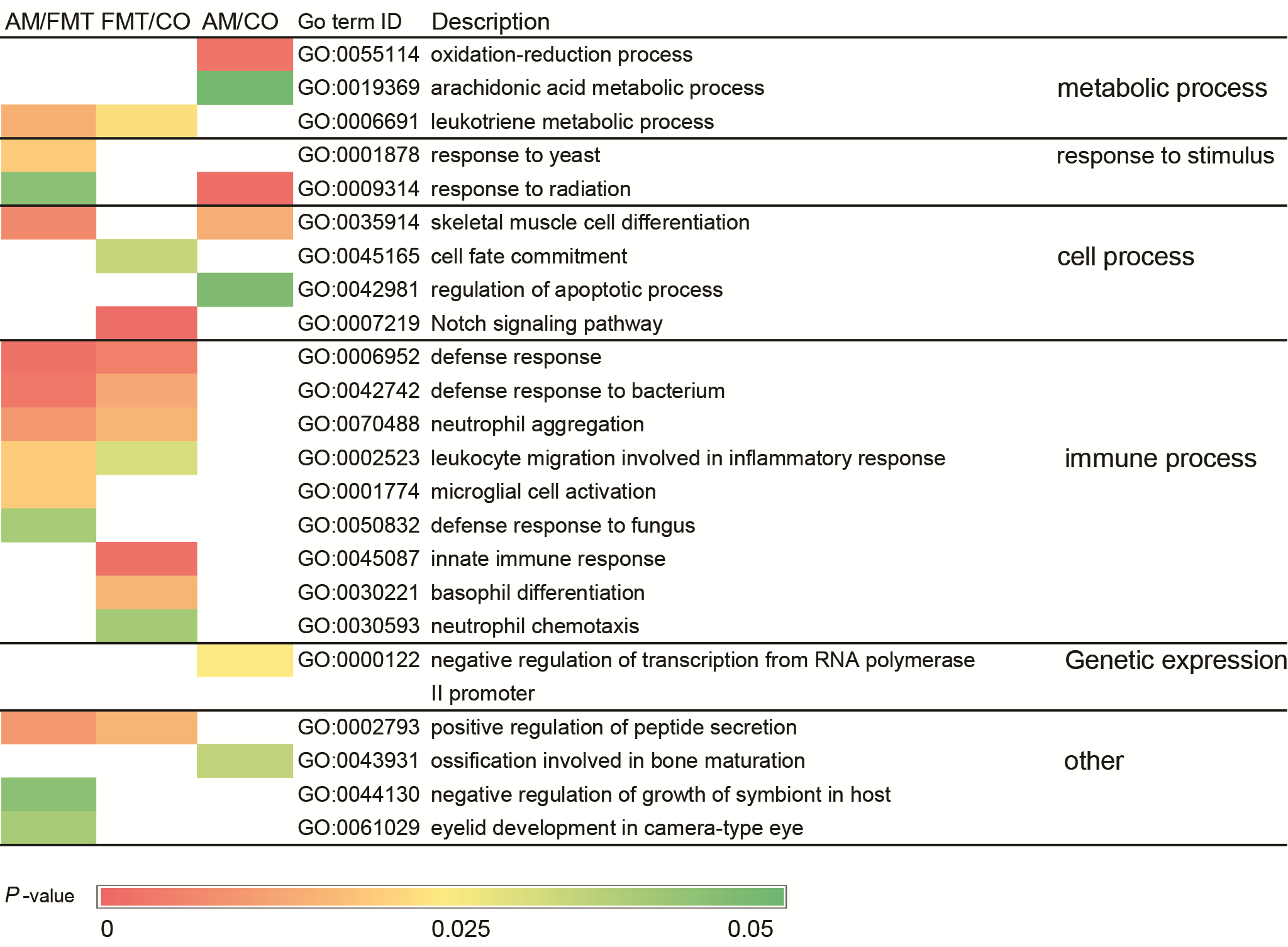
**
